# Circulating cf-miRNA as a more appropriate surrogate liquid biopsy marker than cfDNA for ovarian cancer

**DOI:** 10.1038/s41598-023-32243-x

**Published:** 2023-04-04

**Authors:** Aoife Ward Gahlawat, Tania Witte, Peter Sinn, Sarah Schott

**Affiliations:** 1 Department of Gynaecology and Obstetrics, University Hospital of Heidelberg, Im Neuenheimer Feld 400, 69120 Heidelberg, Germany; 2 National Center for Tumor Diseases (NCT), University Hospital of Heidelberg and German Cancer Research Center (DKFZ), Heidelberg, Germany; 3 Department of Pathology, University Hospital of Heidelberg, Heidelberg, Germany

**Keywords:** Ovarian cancer, Molecular medicine, miRNAs

## Abstract

Ovarian cancer (OC) is an aggressive disease, primarily diagnosed in late stages with only 20% of patients surviving more than 5 years. Liquid biopsy markers have great potential to improve current diagnostic and prognostic methods. Here, we compared miRNAs and DNA methylation in matched plasma, whole blood and tissues as a surrogate marker for OC. We found that while both cfDNA and cf-miRNAs levels were upregulated in OC compared to patients with benign lesions or healthy controls, only cf-miRNA levels were an independent prognosticator of survival. Following on our previous work, we found members of the miR-200 family, miR-200c and miR-141 to be upregulated in both plasma and matched tissues of OC patients which correlated with adverse clinical features. We could also show that the upregulation of miR-200c and -141 correlated with promoter DNA hypomethylation in tissues, but not in plasma or matched whole blood samples. As cf-miRNAs are more easily obtained and very stable in blood, we conclude that they might serve as a more appropriate surrogate liquid biopsy marker than cfDNA for OC.

## Introduction

Ovarian cancer (OC) is a highly malignant disease with a 10-year survival rate of less than 30%, responsible for more than 200,000 deaths worldwide in 2020^1^. Unfortunately, the vast majority of patients present with incurable advanced OC, with a dismal 5-years survival rate of < 20%^2^. In contrast, women diagnosed with early stage disease show an OS of > 90%. The current standard of care for OC is tumour cytoreductive surgery followed by mainly platinum-based chemotherapeutic regimens. However, around half of the patients will develop resistance to chemotherapy or relapse^3^. Therefore, there is a pending need to identify effective biomarkers for early screening, treatment response and prognosis in OC.

The serum tumour marker cancer antigen 125 (CA-125), with a sensitivity of less than 60% in early stage OC and up to 80% in advanced stages^4^ is the current gold standard biomarker for OC diagnosis and monitoring. While CA-125 is an attractive non-invasive marker for OC, the sensitivity and specificity must be improved to implement it as a marker for screening, particularly in early stage disease. Recently, our group has shown that the combination of CA-125 with a panel of seven circulating cell-free microRNAs (cf-miRNAs) could distinguish OC from healthy controls with an AUC of 0.97^5^.

Other studies have also described cf-miRNAs as a promising minimally invasive clinical biomarker for profiling of cancer patients^6–8^. MicroRNAs are non-coding RNAs of 20–25 nucleotides long with the ability to regulate protein coding genes by repressing translation or mRNA degradation. They are transcribed in the nucleus and exported to the cytoplasm, resulting in a mature miRNA^9^. Cf-miRNAs are remarkably stable in body fluids such as plasma, thus making them an ideal non-invasive diagnostic tool for early cancer detection^10–13^. Our group has recently shown, for the first time, that total cf-miRNA levels are an independent prognostic marker for risk stratification in breast cancer^14^.

Likewise, the total amount of cfDNA has been explored as a potential liquid biopsy marker, and has been proposed to stem in varying ratios from DNA released from tumor cells together with DNA fragments from normal cells^15^. With cfDNA, one has the potential to analyze specific alterations coming from the tumor site such as mutations, copy number alterations or aberrant DNA methylation patterns. Since epigenetic aberrations occur early on in tumorigenesis^16^, tumor specific methylation of cfDNA might also be detectable in early stage cancer. However, for early detection, the low abundance of cancer specific cfDNA and corresponding high abundance of background DNA in circulation poses a huge challenge. Until now, only one methylation marker in cfDNA, SEPT9, has been translated to clinical screening with a specificity of 79% and sensitivity of 68% for the detection of colorectal cancer^17^.

Rational combinations of molecular markers in the blood might increase their specificity and sensitivity in diagnostics. Recently, a combination of a panel of DNA mutations and proteins in plasma was able to detect five different cancer types with a sensitivity of 69–98% and a specificity of > 99%^18^. Since miRNA expression can be regulated by aberrant DNA methylation of miRNA promoter sequences in OC^19^, probing for cfDNA promoter methylation and corresponding cf-miRNA abundance may have complementing prognostic value in liquid biopsies.

In this explorative case–control study, we sought out to assess the combination of cf-miRNA and corresponding promoter methylation in OC plasma and tissue samples as potential liquid biopsy markers. Our findings indicate that cf-miRNAs have more potential as a surrogate marker for OC than cfDNA and combinations of other markers such as proteins or mutations should be explored in future.

## Methods

### Sample collection

Before surgery and chemotherapy, women filled in a questionnaire on sociodemographic information and whole blood samples were collected. The cohort is summarised in Supplementary Table 1. Three EDTA tubes (Sarstedt S-Monovette K3E, 1.6 mg EDTA/ml) with 9-ml whole blood were taken from all participants.

### Plasma preparation

Whole blood samples were centrifuged at 1300G for 20 min. The plasma fraction was further processed by high-speed centrifugation at 12,000*g* for 10 min. Samples were immediately stored at − 80.

### Tissue processing and nucleic acid extraction

Fresh frozen tissue sections were obtained from the NCT biobank. Tumor cell content was verified by a pathologist at the Pathology Department. Up to 25 mg of tissue was processed with the Quick-DNA/RNA FFPE Kit (Zymo Research, Freiburg, Germany) for simultaneous extraction of genomic DNA and total RNA, including miRNAs. Nucleic acid concentration and purity was confirmed with the NanoDrop™ 1000 UV/Vis-Spectralphotometer 3.3 (peqLab, Erlangen, Germany).

### miRNA isolation from plasma

Circulating miRNAs were isolated from 300 µl thawed plasma using the NucleoSpin miRNA Plasma kit (Macherey–Nagel, Düren, Germany) according to the manufacturer’s procotol. Total miRNAs were quantified using the Qubit microRNA Assay Kit and the Qubit Fluorometer 3.0 (Thermo Fisher Scientific, Massachusetts, USA).

### qRT-PCR

For miRNA extracted from plasma, 2 µl was synthesized to cDNA using the LNA miRNA RT kit (Qiagen). For total RNA extracted from tissue, 10 ng was synthesized to cDNA using the LNA miRNA RT kit. Individual miRNAs were amplified and quantified using LNA specific primers and the primaQUANT 2 × qPCR-CYBR-Green-Blue-MasterMix (Steinbrenner, Germany) according to the manufacturer’s protocol on the qTOWER instrument (Analytical Jena, Germany). Two replicates were performed for each sample. For plasma samples, the geometric mean across the cohort was used to calculate miRNA expression^20^. For tissue samples, relative expression was calculated using the U6 snoRNA as a reference.

### cfDNA isolation

Plasma was thawn and cfDNA was isolated from up to 2 ml using the NucleoSnap cfDNA kit (Macherey-Nagel, Düren, Germany) according to the manufacturer’s procotol. cfDNA was quantified using the Qubit dsDNA HS Assay kit and the Qubit Fluorometer 3.0 (Thermo Fisher Scientific). DNA size distribution was assessed on the Bioanalyzer instrument using the DNA High Sensitivity Kit (Bioanalyzer, CA, USA).

### DNA methylation analysis

For bisulfite conversion, 500 ng of tissue genomic DNA or 5–100 ng cfDNA was converted using the EZ DNA Methylation-Gold™ kit (Zymo Research, Freiburg; Germany). Subsequent PCR amplification was performed using HotStarTaq Plus DNA Polymerase kit (Qiagen, Hilden, Germany) according to manufacturer’s instructions incorporating the T7 promoter sequence as listed in Supplementary Table 1. PCR products were verified by electrophoresis with a 1% agarose gel. According to the instructions of Sequenom MassARRAY EpiTyper Assay the PCR products were subjected to alkaline phosphatase treatment followed by in vitro transcription and RNaseA cleavage to result in specific fragmentation. The obtained fragments were subjected to matrix-assisted laser desorption/ionization time-of-flight mass-spectrometry (MALDI-TOF MS). Results were exported from the MassARRAY instrument with EpiTyper v1.0 software. Unless otherwise stated, DNA methylation values were calculated as the average methylation of all CpG sites within each PCR product.

### Statistical analysis

Significance between groups was calculated by a nonparametric Mann Whitney test using GraphPad prism version 8.0. Kaplan–Meier survival plots for single markers were also computed with GraphPad prism, using the Log-rank model for significance. All other analyses were performed using R version 4.1.2. In order to compute univariate hazard ratios, a Cox proportional hazards regression model^21^ was fitted for each parameter separately with the respective overall or progression-free survival time as a dependent variable and the respective marker as a single covariate. Multivariate hazard ratios are stemming from a multivariate Cox proportional hazards regression model, where all parameters simultaneously were used as covariates when fitting the Cox models for overall or progression-free survival. For all Cox models, p values for the null hypothesis that the hazard ratio equals to 1 were derived by means of a standard Wald test. The REMARK (Reporting Recommendations for Tumor Marker Prognostic Studies) guidelines were implemented to report results^22^.

### Ethics approval and consent to participate

Ovarian cancer (OC) patients (n = 72), women who were treated for unknown pelvic mass and healthy volunteers with no known conditions (n = 53) were recruited at the University Hospital Heidelberg, Germany and at the National Center for Tumor Diseases (NCT), Heidelberg, Germany, between May 2015 and August 2018. All participants provided written informed consent. The study was approved by the ethics committee of the University of Heidelberg (S-046/2018, S-266/2011, S-393/2019) in accordance with good clinical practice guidelines, national laws and the Declaration of Helsinki.

## Results

### Study population

**Figure 1:**
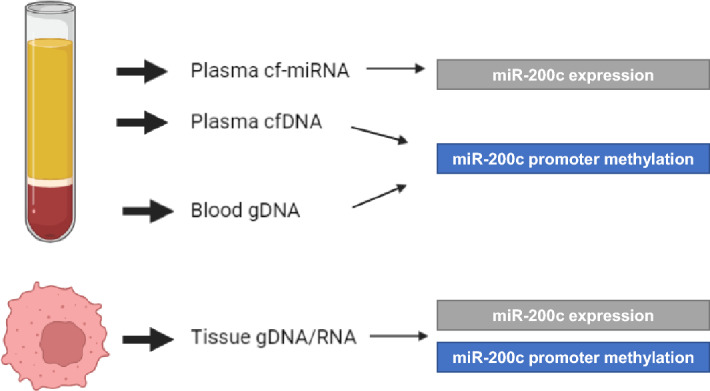
Study overview. Plasma cf-miRNA and cf-DNA was isolated from 125 study participants and analysed for miR-200c expression and promoter methylation analysis. Genomic DNA was obtained from whole blood (n = 110). Genomic DNA and RNA were simultaneously isolated from matched tissues (n = 46). This figure was created with Biorender.com and exported under a paid subscription.

Plasma and whole blood from EDTA tubes as well as fresh frozen tissues were used in this study as outlined in Fig. 1. Patients were recruited at the Heidelberg University Hospital between May 2015 and August 2018. The cohort characteristics are outlined in Supplementary Table 1.

## Total levels of circulating cfDNA and cf-miRNAs are globally upregulated in OC

**Figure 2:**
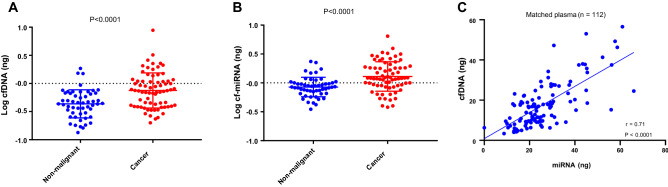
Circulating cfDNA and cf-miRNAs are upregulated in OC. Total cfDNA and cf-miRNAs were measured in plasma. Dot plots depict the total levels (log transformed) of cfDNA (**A**) and cf-miRNAs (**B**) in a group of healthy controls and patients with benign lesions (non-malignant) and OC cases. Significance is calculated by a Mann Whitney test.

Since circulating nucleic acids are proposed to be released from tumors and can serve as surrogate markers, we first assessed whether OC patients have more cfDNA and cf-miRNA amounts compared to patients with benign lesions or healthy women with no known malignancies. cfDNA and cf-miRNA was isolated from the matched plasma samples, using independent isolation methods. Because there was no difference between the healthy controls and benign group (Supp Fig. 1), we decided to pool these two groups hereby called “non-malignant”. We also log transformed the data due to abnormal distribution and found a significant increase in the amounts of both cfDNA and cf-miRNA in OC cases (Fig. 2A,B). Remarkably, the total amount of circulating cfDNA and miRNA was positively correlating in matched plasma samples (Fig. 2C).

### Circulating cf-miRNA but not cfDNA is an independent marker of survival in OC

**Figure 3:**
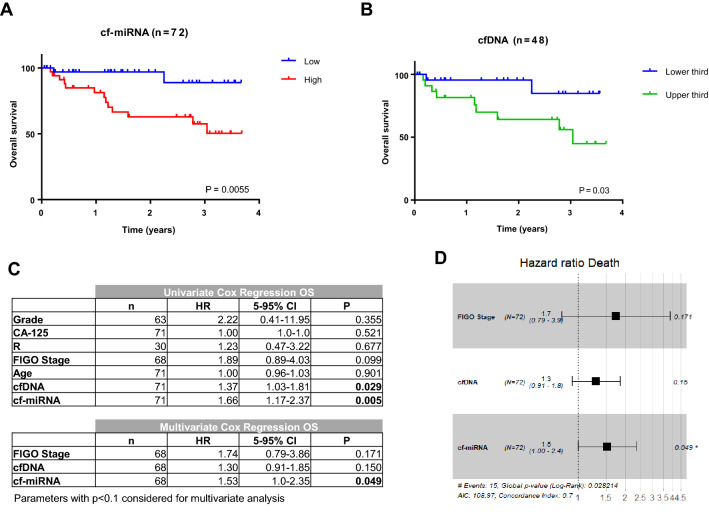
Circulating miRNAs are prognostic biomarkers in OC. Kaplan–Meier curves show a significant correlation between OS and miRNA levels (**A**) and cfDNA levels (**B**) cohorts. Univariate and multivariate cox regression analysis was performed (**C**) and multivariate analysis is displayed as a forest plot (**D**).

Our group has recently shown that total levels of cf-miRNAs have prognostic value in both breast^14^ and OC^5^. Because cfDNA levels were similarly increased as cf-miRNA levels in OC, we hypothesized that the levels of cfDNA might also be associated with patient outcome. To this end, we dichotomized the patients according to the median levels of circulating DNA or miRNA and could reproduce in the reduced cohort, where plasma was available for both analyses (n = 72), that cf-miRNA levels significantly associated with survival (Fig. 3A; P = 0.005). However, levels of cfDNA were not associated with survival using the same approach of dichotomization (Supp Fig. 2A) but only when we split the data into 3 sub-groups in a reduced number of patients (Fig. 3B; P = 0.03). Next, we performed univariate and multivariate cox regression analysis using Grade, CA-125, R status, FIGO stage and age as categorical variables. Strikingly, only levels of cfDNA and cf-miRNA were significant predictors of survival in univariate analysis. Finally, in multivariate analysis including FIGO stage, cfDNA and cf-miRNA levels, only cf-miRNA remained as a significant independent predictor of survival in this cohort (Fig. 3C,D). Neither cfDNA nor cf-miRNA were significantly associated with progression-free survival (Supp Fig. 2).

### Circulating miR-200c and miR-141 are surrogate markers for OC and correlate with adverse clinical features

**Figure 4:**
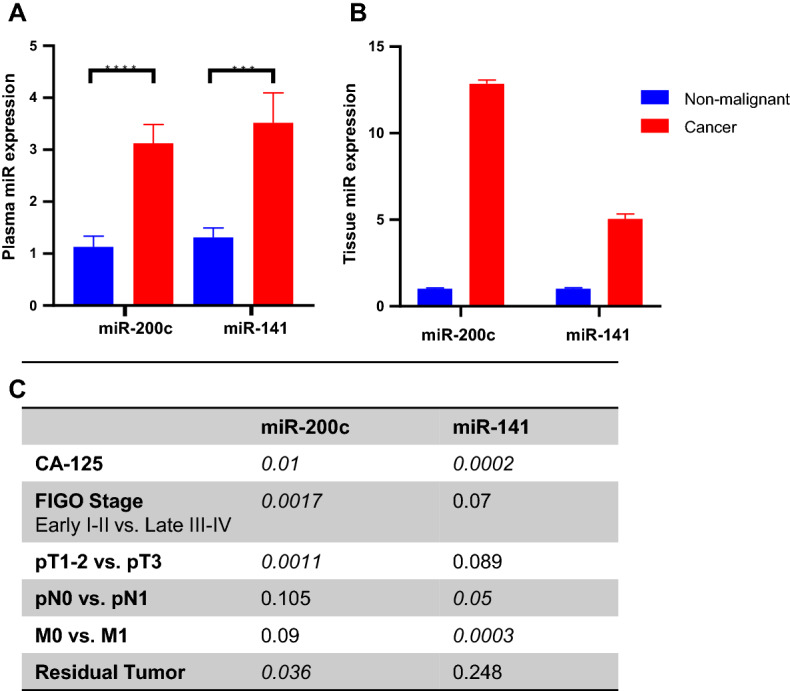
Circulating miR-200c and miR-141 are potential surrogate markers for OC. Expression levels of circulating miR-200c and -141 are significantly upregulated in OC cases compared to controls, as depicted by box plot with SEM (**A**). Both miR-200c and miR-141 expression is upregulated in a panel of OC tissues compared to benign tissues (**B**). Significance is calculated by a Mann Whitney test and depicted as ***P < 0.001 and ****P < 0.0001. The table below shows the correlation of circulating miRNA expression with clinical features of OC.

Next, we were interested to know which miRNAs were contributing to the increase of total miRNA levels in OC patients. Previously, our group identified a signature of 7 miRNAs which could serve as diagnostic markers for OC^5^. One of these candidates, miR-200c has already been documented to be upregulated in OC^23^. miR-200c belongs to the miR-200 miRNA family which have repeatedly been described as modulators of the metastatic cascade in cancer. Since miR-200c is transcribed together and generally co-expressed with miR-141, we analysed the expression of both miRNAs in OC plasma and a subset of matched tissues by qRT-PCR. Both miR-200c and miR-141 were significantly increased in both plasma and tissues in OC (Fig. 4A,B). Additionally, increased circulating levels of miR-200c and -141 significantly associated with a number of adverse clinical features, highlighting their potential as surrogate markers for OC (Fig. 4C).

### Promoter methylation of miR-200c and miR-141 correlates with expression in tissues but not in plasma

**Figure 5:**
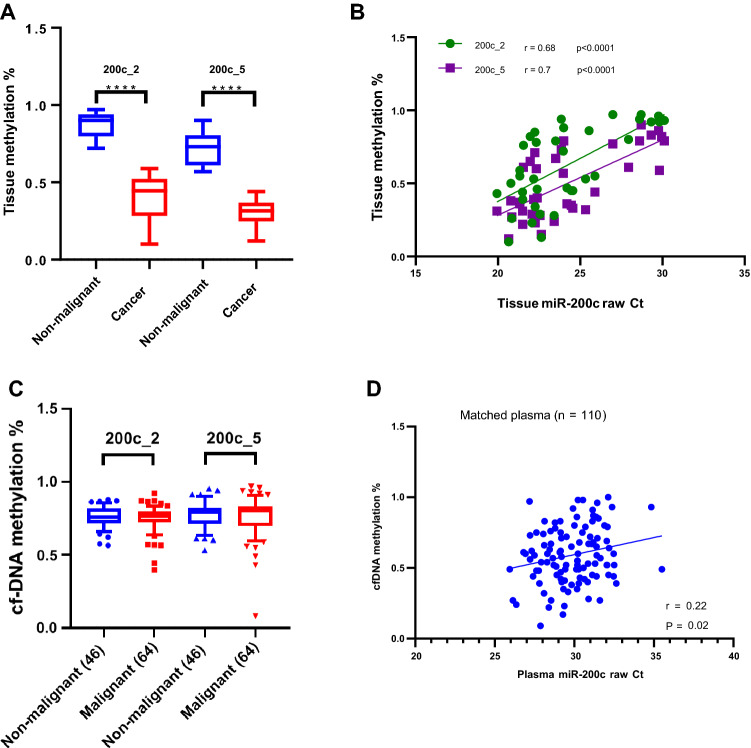
miR-200c expression correlates with promoter methylation in OC tissues but not in cfDNA. Quantitative DNA methylation analysis of the miR-200 promoter in a panel of non-malignant and cancer tissues is depicted as percentage where ****P < 0.0001 (**A**). DNA methylation correlated with matched miRNA expression, plotted as raw Ct values, in corresponding tissues (n = 38) (**B**). Quantitative DNA methylation analysis of the miR-200 promoter in cfDNA of non-malignant and malignant OC (**C**) and one representative CpG site *P < 0.05 (**D**). The correlation between cfDNA methylation and miRNA expression is shown (**E**).

Since the bulk of circulating cfDNA is likely not tumor specific, we sought to look for a more specific parameter that could be used in combination with cf-miRNAs as a surrogate marker for OC. We hypothesized that the miR-200 family might be epigenetically regulated in OC, as concluded by a recent meta-analysis^24^. The miR-200 family consists of five miRNAs, miR-200a, miR-200b and miR-429; transcribed on chromosome 1 and miR-200c and miR-141; transcribed on chromosome 12. With a difference of one base pair in the seed region, the miR-200 family have the potential to target thousands of mRNAs which can have a profound impact in disease^25^. We utilized the quantitative MassArray technology^26^ to analyse DNA methylation of two regions: 200c_2 and 200c_5 in the upstream promoter region of miR-200c on chromosome 12 (Supplementary Fig. 3a) in the same set of tissues where we had measured miRNA expression (Fig. 4B). We found a highly significant promoter hypomethylation in genomic DNA of tumor tissues compared to benign in both regions (Fig. 5A) which significantly correlated with miR-200c (over)expression in the matched tissue samples (Fig. 5B). Next, we sought out to investigate DNA methylation in cfDNA samples. 110 samples of 125 had sufficient quality to undergo MassArray analysis. In contrast to tumor tissues, we observed no significant differences in miR-200c promoter methylation in plasma samples (Fig. 5C). Overall, the variation in methylation in plasma was much broader compared to the tissues. Of interest, a mild but significant correlation between matched plasma cfDNA methylation and miRNA expression was observed (Fig. 5D), indicating that at least in part, the cfDNA methylation profile reflected that of primary tumors. Direct comparison of matched tissue and cfDNA revealed that the majority of patients with hypomethylated DNA from tissue had hypermethylated cfDNA (Supplementary Fig. 3B), indicating a strong influence of background signal in the plasma. Promoter methylation analysis of genomic DNA from whole blood (n = 143) confirmed this observation, however, in contrast to cfDNA (Supplementary Fig. 3C) and there was no correlation between whole blood methylation and miRNA expression (data not shown). In conclusion, methylation of the miR-200c promoter in cfDNA was partly reflective of tumor tissue methylation in OC.

## Discussion

In summary, we presented one of the only studies in OC where cf-miRNA, cfDNA, whole blood and matched tissue samples have been simultaneously investigated. We have again demonstrated the potential of cf-miRNA levels as an independent prognostic marker for survival compared to cfDNA. We have also shown that two circulating miRNAs have potential as surrogate markers in OC. We verified one possible biological mechanism of miRNA regulation by DNA methylation in matched tumor tissues and partly in cfDNA. Our results indicate that circulating miRNAs might be a more suitable liquid biopsy marker than cfDNA in OC, and combinations with other markers such as proteins or mutations should be explored in the future to evaluate options for disease diagnosis and therapy monitoring.

While levels of circulating cfDNA and mutational load have been readily uptaken in the clinic for disease monitoring, circulating cf-miRNAs are still far from implementation. We believe that cf-miRNAs are more appropriate as surrogate markers for OC and an ideal plasma biomarker in comparison to total cfDNA for a number of reasons. First of all, previous work from our lab demonstrated that miRNAs, including miR-200c, are highly stable in blood^13^ and can be obtained from as little as 200 µl plasma. They are easily detectable in both healthy and diseased individuals by simple amplification methods such as qPCR. On the contrary, cfDNA is hardly detectable in healthy individuals and the amount of blood required for analysis is at least 2 ml. We have shown, for the first time, that cf-miRNA is more representative of corresponding tissues compared to cfDNA methylation. The miR-200 promoter was significantly hypomethylated in a panel of tissues which strongly correlated with miRNA expression. On the other hand, no differences on general methylation were observed in cfDNA, and in a subset of patients with matched tissues, we observed a general increased methylation of corresponding cfDNA. One explanation for this may be that the signal is diluted due to the influence of normal circulation, probably coming from blood cells. Indeed, whole blood methylation analysis confirmed the results in cfDNA. Nevertheless, we did see a trend toward hypomethylation at specific CG sites, which partly correlated with miRNA expression in corresponding plasma.

Overexpression of the miRNA-200 family has already been well described in OC^27^. In line with our study, miR-200a/b/c were elevated in OC patient serum (n = 70) which associated with disease progression, advanced stage and metastasis^28^. Another recent study of ascites, the fluid released from the surrounding pelvic region in OC patients, also found upregulation of the miR-200 family^29^. We have demonstrated that DNA methylation of the upstream miRNA promoter region associates with miR-200c expression in OC tissues and partly in plasma. Similarly, a recent meta-analysis on the TCGA cohort revealed a correlation between promoter methylation and miR-200 expression in more than 500 OC patients^24^. While most studies describe the miR-200 family as tumor suppressive miRNAs, downregulated in cancer, some studies have had similar findings to ours. For example in pancreatic cancer, hypomethylation and upregulation of miR-200a and 200b led to an epithelial-mesenchymal transition (EMT) transition^30^. While we did not directly characterize EMT in our study, we also observed higher levels of circulating miR-200c and -141 in patients with lymph node infiltration and metastatic disease. In addition to EMT, epigenetic regulation of the miR-200 family has been attributed to drug resistance in cancer. Shindo and colleagues demonstrated that miR-200b,a and miR-429 were downregulated in cisplatin resistant bladder cancer which associated with CpG island hypermethylation^31^. As platinum resistance is also a major contributor in OC progression^32^ circulating miR-200 might also have potential in disease monitoring.

While, the field of liquid biopsy is currently placing a large emphasis on cfDNA, we could show that a small panel of cf-miRNAs are more likely to reflect the tumor status. Few studies until now have been able to show a significant impact of cfDNA methylation in OC. Recently, Giannopoulou et al. found a moderate correlation between ESR1 methylation in OC primary tumors and matched cfDNA^33^. The technology used to assess cfDNA methylation should also be considered. Most studies until now have used PCR based methylation assays which are not very accurate and introduce PCR bias. We have utilized the quantitative MassARRAY technology based on mass spectrometry^26^ which has the major advantage of accurately quantifying methylation levels. Another technical challenge with methylation analysis is that most methods require a bisulfite conversion step, which can degrade up to 96% of DNA^34^. A limitation of our work is that we focused on just one miRNA promoter. A whole genome methylation screening should be implemented to identify novel markers. Although this is difficult due to the low amount of cfDNA available, one study until now has performed methylome screening in a large panel of tumor tissues and serum from OC. They found that methylation of just three genes could identify 90% of cases and was predictive of therapy response^35^. In future, we may look to analyse the same set of markers in corresponding tissues and plasma. Similarly to our results, the authors also reported a discrepancy between cfDNA and tumor tissue methylation, possibly due to contamination of blood cells in the plasma. Another angle could be to combine miRNA markers with mutation status in cfDNA. Recently, this approach in combination with protein markers has shown great promise in OC^18^.

One strength of our study is the same material (matched plasma miRNA, cfDNA, whole blood and tissue) was available from the same donors for analysis, albeit only from one cohort and not for all samples. For miRNA analysis, the number of individuals analysed across independent cohorts was considerably large for OC. No studies until now have utilized cf-miRNA levels as a biomarker, or compared this to cfDNA from the same donor. Even though the correlation of both nucleic acids was significant, miRNA levels surpassed cfDNA with the association of clinical features. In particular, miRNA levels were strongly associated with OS, which we have previously shown for both OC^5^ and breast cancer^14^ which was not the case for cfDNA. This finding suggests that cf-miRNAs may be even more informative than traditional biopsies as prognostic markers. A potential explanation for this may be due to the protection of miRNAs in circulation. While DNA fragments are released from tumors or blood cells, miRNAs are either packaged into extracellular vesicles or exported from cells in a protein complex. These mechanisms can protect cf-miRNAs from degradation and may explain why their specificity for cancer is better retained than for cfDNA. Further studies should be done to elaborate on this point.

The introduction of novel targeted therapies, namely the anti-angiogenic bevacizumab during the course of our sample collection may have also impacted the prognosis of our cohort^36^ and thus our conclusions related to cf-miRNA levels. One recent study in breast cancer tissues, found a number of changes in miRNA expression in patients undergoing neoadjuvant therapy with bevacizumab^37^. We are not aware of any such studies regarding cf-miRNA and we did not have access to longitudinal samples in our study to analyse this.

In conclusion, our data support practicability and clinical relevance of circulating miRNAs as liquid biopsy markers and propose prognostic potential of circulating miR-200c and miR-141 for OC. Ultimately, we describe a link between miR-200c overexpression and miR-200c promoter hypomethylation in OC tissue, which is partly reflected by cfDNA.

## Supplementary Information

Supplementary Information.

## Data Availability

The datasets used and/or analysed during the current study available from the corresponding author on reasonable request.
