# Supplementary material for: Circulating cf-miRNA as a more appropriate surrogate liquid biopsy marker than cfDNA for ovarian cancer
Source: Sci Rep. 2023 Apr 4;13:5503. doi: 10.1038/s41598-023-32243-x (PMC10073086; doi:10.1038/s41598-023-32243-x)

Supplementary Table 1: Cohort characteristics

		Non-malignant	Cancer
n		53	72
Mean age (range)		58.7 (25-85)	61 (21-83)
CA-125 (n)	< 35 U/ml	45	7
	> 35 U/ml	7	58
**Histology**			n
	Serous		55
	Non-serous		17
**FIGO Stage**	I - II		12
	III - IV		53
**Grade**	1 and 2		11
	3		52
**Tumor size**	T1-T2		15
	T3		40
**Lymph nodes**	N0		24
	N1+		23
**Metastasis**	M0		7
	Local		49
	M1 distant		12
**Residual Tumor**	R0		20
	R1		9

Supplementary Table 2 – Primer sequences for promoter methylation

Amplicon	Primer	Sequence
200c_2	miR200c.2_10F	aggaagagagGGGGTTTAGGTAAGGAGTTTG
	miR200c.2_T7R	cagtaatacgactcactatagggagaaggctCTCTACAATCCCAAACTCAAC
200c_5	miR200c.5_10F	aggaagagagGTTGAGTTTGGGATTGTAGAG
	miR200c.5_T7R	cagtaatacgactcactatagggagaaggctCTTCCTAATAAACCCTACTAC

Supp Figure 1: Levels of cfDNA and cf-miRNA in plasma

Depicted as bar charts showing control, benign and cancer groups, subdivided by tumor pathology status

A B

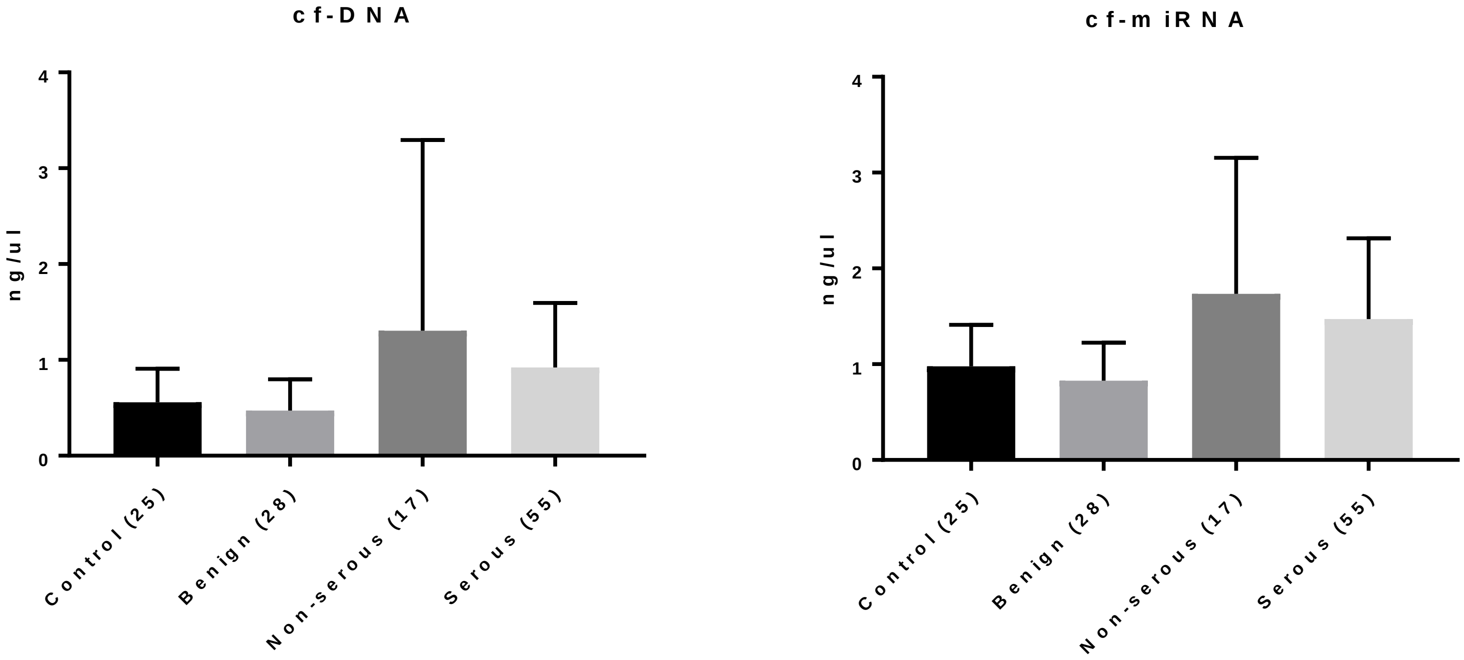

Supp Figure 2: Kaplain-Meier analysis

Overall survival and Progression free survival plotted by median levels of cfDNA or cf-miRNA (A-C), uni and multi-variate Cox regression analysis (D) and hazard ratio depicted as forest plot (E)

A

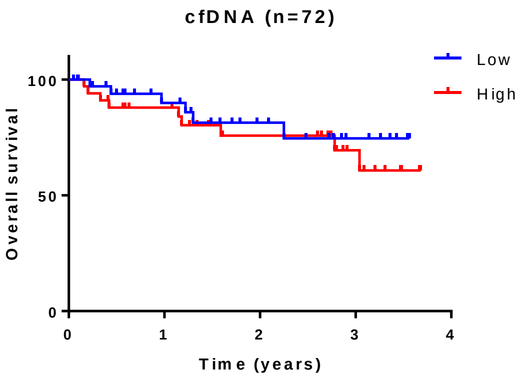

B

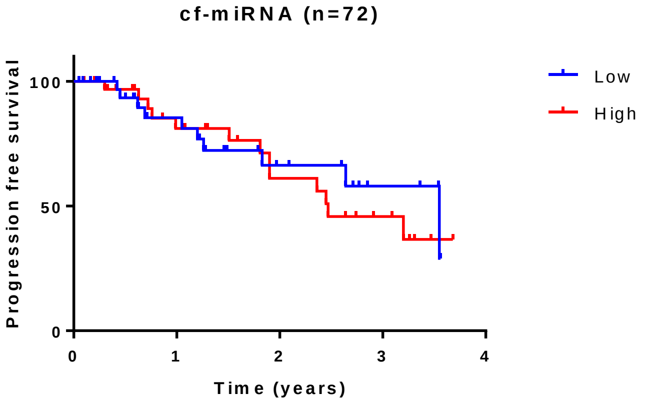

C

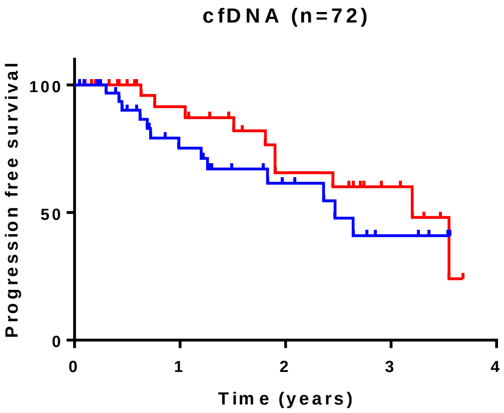

D

	Univariate Cox Regression PFS			
	n	HR	5-95% CI	p
**Grade**	63	0.63	0.29-1.38	0.251
**CA-125**	71	1.00	1.0-1.0	0.896
**R**	30	2.22	1.02-4.86	**0.045**
**FIGO Stage**	68	1.80	0.99-3.26	0.054
**Age**	71	1.01	0.98-1.04	0.686
**cf-miRNA**	71	1.09	0.68-1.74	0.729
**cfDNA**	71	0.69	0.34-1.38	0.293

	Multivariate Cox Regression PFS			
	n	HR	5-95% CI	p
**Grade**	61	2.12	0.37-12.27	0.401
**FIGO Stage**	61	1.69	0.76-3.74	0.195
**cfDNA**	61	1.34	0.97-1.86	0.074

Parameters with p<0.3 considered for multivariate analysis

E

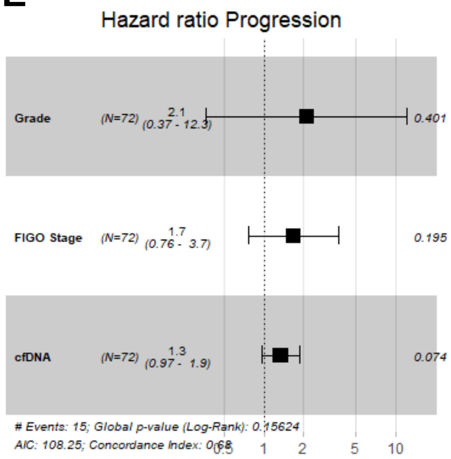

Supplementary Figure 3: Methylation of miR-200 promoter

The regions assessed by mass array on chromosome 12 are depicted (A), the comparision of methylation in tissue and matched cfDNA (B) and whole blood methylation analysis (C)

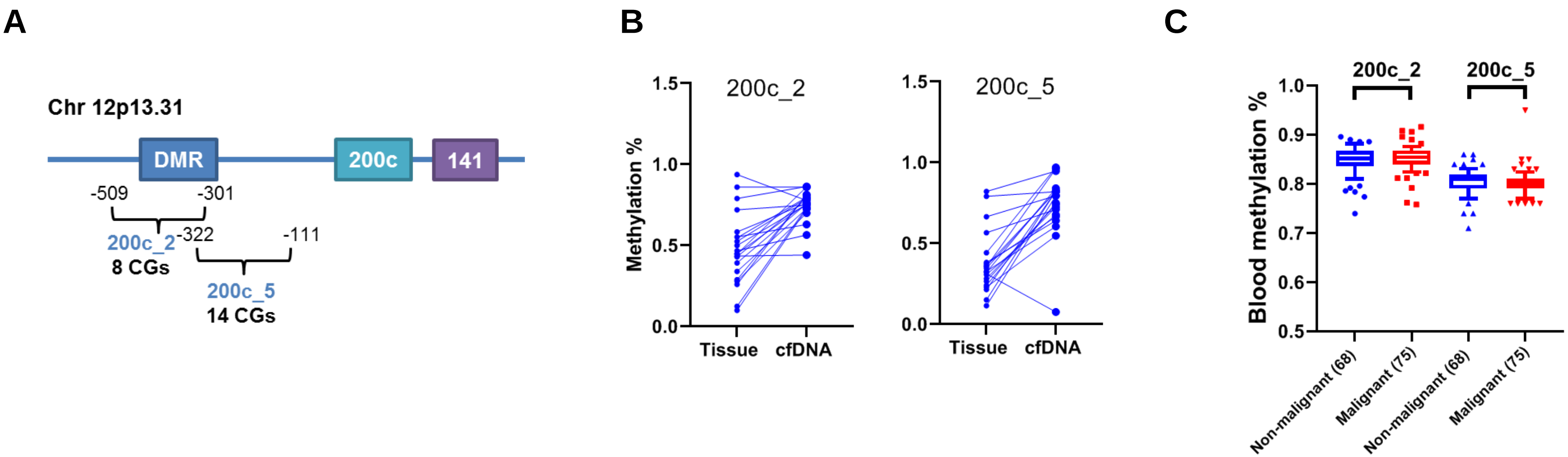

Supplement: Supplementary file 1 — Supplementary Information. [file 41598_2023_32243_MOESM1_ESM.pdf]
